# Induction of labour versus expectant monitoring in women with pregnancy induced hypertension or mild preeclampsia at term: the HYPITAT trial

**DOI:** 10.1186/1471-2393-7-14

**Published:** 2007-07-27

**Authors:** Corine M Koopmans, Denise Bijlenga, Jan G Aarnoudse, Erik van Beek, Dick J Bekedam, Paul P van den Berg, Jan M Burggraaff, Erwin Birnie, Kitty WM Bloemenkamp, Addi P Drogtrop, Arie Franx, Christianne JM de Groot, Anjoke JM Huisjes, Anneke Kwee, Saskia le Cessie, Aren J van Loon, Ben WJ Mol, Joris AM van der Post, Frans JME Roumen, Hubertina CJ Scheepers, Marc EA Spaanderman, Rob H Stigter, Christine Willekes, Maria G van Pampus

**Affiliations:** 1 Department of Obstetrics and Gynaecology, University Medical Centre Groningen, The Netherlands; 2 Department of Obstetrics and Gynaecology, Martini Hospital Groningen, The Netherlands; 3 Department of Social Medicine, Academic Medical Centre Amsterdam, The Netherlands; 4 Department of Obstetrics and Gynaecology, Sint Antonius Hospital Nieuwegein, The Netherlands; 5 Department of Obstetrics and Gynaecology, Onze Lieve Vrouwen Gasthuis Amsterdam, The Netherlands; 6 Department of Obstetrics and Gynaecology, Scheper Hospital Emmen, The Netherlands; 7 Department of Obstetrics and Gynaecology, Leiden University Medical Centre, The Netherlands; 8 Department of Obstetrics and Gynaecology, Twee Steden Hospital Tilburg, The Netherlands; 9 Department of Obstetrics and Gynaecology, Sint Elisabeth Hospital Tilburg, The Netherlands; 10 Department of Obstetrics and Gynaecology, Medical Centre Haaglanden Den Haag, The Netherlands; 11 Department of Obstetrics and Gynaecology, Gelre Hospital Apeldoorn, The Netherlands; 12 Department of Perinatology and Gynaecology, University Medical Centre Utrecht, The Netherlands; 13 Department of Mediacl Statistics, Leiden University Medical Centre, The Netherlands; 14 Department of Obstetrics and Gynaecology, Academic Medical Centre Amsterdam, The Netherlands; 15 Department of Obstetrics and Gynaecology, Maxima Medical Centre Veldhoven, The Netherlands; 16 Department of Obstetrics and Gynaecology, Atrium Medical Centre Heerlen, The Netherlands; 17 Department of Obstetrics and Gynaecology, University Medical Centre Nijmegen, The Netherlands; 18 Department of Obstetrics and Gynaecology, Deventer Hospital, The Netherlands; 19 Department of Obstetrics and Gynaecology, University Hospital Maastricht, The Netherlands

## Abstract

**Background:**

Hypertensive disorders, i.e. pregnancy induced hypertension and preeclampsia, complicate 10 to15% of all pregnancies at term and are a major cause of maternal and perinatal morbidity and mortality. The only causal treatment is delivery. In case of preterm pregnancies conservative management is advocated if the risks for mother and child remain acceptable. In contrast, there is no consensus on how to manage mild hypertensive disease in pregnancies at term. Induction of labour might prevent maternal and neonatal complications at the expense of increased instrumental vaginal delivery rates and caesarean section rates.

**Methods/Design:**

Women with a pregnancy complicated by pregnancy induced hypertension or mild preeclampsia at a gestational age between 36^+0 ^and 41^+0 ^weeks will be asked to participate in a multi-centre randomised controlled trial. Women will be randomised to either induction of labour or expectant management for spontaneous delivery. The primary outcome of this study is severe maternal morbidity, which can be complicated by maternal mortality in rare cases. Secondary outcome measures are neonatal mortality and morbidity, caesarean and vaginal instrumental delivery rates, maternal quality of life and costs. Analysis will be by intention to treat. In total, 720 pregnant women have to be randomised to show a reduction in severe maternal complications of hypertensive disease from 12 to 6%.

**Discussion:**

This trial will provide evidence as to whether or not induction of labour in women with pregnancy induced hypertension or mild preeclampsia (nearly) at term is an effective treatment to prevent severe maternal complications.

**Trial Registration:**

The protocol is registered in the clinical trial register number ISRCTN08132825.

## Background

Pregnancy induced hypertension and preeclampsia are common complications of pregnancy [1]. In many cases, the clinical presentation is mild, consisting only of mild hypertension and/or mild proteinuria at term. In other cases however, severe maternal and fetal complications such as eclampsia, abruptio placentae, preterm delivery, the Hemolysis Elevated Liver enzymes and Low Platelet count syndrome (HELLP), fetal growth restriction or even intra-uterine fetal death may occur. Hypertensive disorders in pregnancy make a major contribution to maternal and neonatal mortality. In the Netherlands, hypertensive disorders in pregnancy are the largest single cause of maternal mortality [2].

Approximately 10% to 15% of all pregnancies are complicated by hypertensive disorders. The vast majority of these cases occur after 32 weeks. The only causal treatment of the disease is delivery. In case of preterm pregnancies (28–34 weeks gestational age) complicated by preeclampsia expectant monitoring is advocated to increase the chance of fetal maturity, as long as the risks for the mother remain acceptable [3-5]. Expectant management reduces neonatal complications and duration of neonatal stay in the intensive care unit in preterm pregnancies and is not associated with an increase in maternal complications [4,5].

In case of pregnancy induced hypertension or preeclampsia at term, the situation is different from preterm disease. In women with mild preeclampsia complications such as abruptio placenta and small for gestational age are similar to normotensive pregnancies. It is unclear whether in this situation expectant management is beneficial for the mother and her baby, since evidence is lacking. Despite this lack of evidence delivery is often recommended because of the unpredictability of the disease [4,6]. Recent observational studies indicate that the onset of mild gestational hypertension or mild preeclampsia at or near term is associated with minimal to low maternal and neonatal morbidity [6-8]. Despite the lack of evidence that would justify intervention, many obstetricians induce labour in women at term with pregnancy-induced hypertension or preeclampsia. Such a policy may increase the risk of assisted vaginal delivery and caesarean section, thus generating additional morbidity and costs [9-11]. On the other hand, expectant management might lead to severe pregnancy complications like eclampsia, severe hypertension, HELLP syndrome, organ failure or an adverse neonatal outcome.

Data from the Dutch National Obstetric Registration from 2002 showed that the yearly number of patients with hypertension (blood pressure [BP] diastolic above 90 mmHg) without proteinuria at term is 17.000. Moreover, there are 2.000 women with preeclampsia at term. The lack of consensus is demonstrated by the fact that in 9.000 women with pregnancy induced hypertension or preeclampsia labour was induced, whereas labour started spontaneously in 10.000 women. Moreover, national data indicate no impact of induction of labour on neonatal outcome. In 2002 and 2003, the rate of babies born with a 5-minute Apgar score below 7 was 1.3% among women that delivered after a spontaneous onset of labour, versus 1.6% among women in whom labour was induced (OR 1.2 95% CI 1.0 to 1.5). After adjustment for potential confounders such as fetal weight, proteinuria and diastolic blood pressure, this difference became statically insignificant despite the analysis of over 35.000 patients (OR 1.1 95% CI 0.98 to 1.2). Since this equivalence is also expected from the pathophysiological background of the problem as well as from the medical literature, we anticipate no differences in neonatal outcome between both strategies.

Data from the Dutch National Obstetric Registration from Januari 2000 until Januari 2005 show that 38.170 nullipara had a singleton pregnancy in cephalic presentation complicated with pregnancy induced hypertension or preeclampsia. In 18.012 women labour started spontaneously, whereas in 18.810 labour was induced. The non-elective caesarean section rate among women in whom labour started spontaneously was 14% and among women in whom labour was induced this rate was 22% (OR 1,7 95% CI 1,6 to 1,8). The vaginal instrumental delivery rates among these groups were 28% and 24% (OR 0,88 95% CI 0,84 to 0,93).

At present, there is no evidence on the effectiveness and efficiency of induction of labour in women with pregnancy induced hypertension or mild preeclampsia (nearly) at term as compared with expectant management with close monitoring. In post term women and women with ruptured membranes at term, randomised trials have indicated that induction of labour does not increase the instrumental delivery rate [12,13]. However, the fact that the women were post term, might implicate that myometrial gapjunctions facilitating effective contractions were present [12]. These data can not be extrapolated to women who are (nearly) at term with pregnancy induced hypertension or preeclampsia.

In view of this clinical dilemma, we propose a randomised clinical trial in which a policy of induction of labour, if necessary preceded by artificial cervical ripening, is compared with a policy of careful expectant monitoring in women with pregnancy induced hypertension or mild preeclampsia (nearly) at term. At present – to our knowledge – no clinical study has been published or undertaken to investigate this issue.

## Methods/Designs

### Aims

The aim of this study is to investigate whether planned induction of labour compared with expectant management in women with pregnancy induced hypertension or mild preeclampsia at term will reduce severe maternal morbidity. We hypothesize that induction of labour will reduce maternal morbidity and mortality. The study will also provide insight on whether induction of labour in women with pregnancy induced hypertension or preeclampsia (nearly) at term will reduce costs and improve quality of life as compared to expectant monitoring.

The proposed research concerns a multi-centre randomised controlled clinical trial in women who have pregnancy induced hypertension or mild preeclampsia at gestational ages between 36^+0 ^and 41^+0 ^weeks. This study is set in a national Obstetric Research Consortium, in which 40 obstetric clinics in the Netherlands collaborate. Approximately 40 clinics, including academic hospitals, non-academic teaching hospitals and non-teaching hospitals will participate in this trial.

### Participants/Eligibility criteria

Patients 18 years of age or older will be eligible if they have pregnancy induced hypertension or mild preeclampsia at a gestational age between 36^+0 ^and 41^+0 ^weeks of gestation. A diagnosis of pregnancy induced hypertension is made in case the diastolic BP is equal to or above 95 mmHg at two occasions at least six hours apart in a woman who was normotensive at the start of pregnancy until week 20 of gestational age. A diagnosis of mild preeclampsia is made in case the diastolic BP is above 90 mmHg and there exists a proteinuria > 300 mg total protein in a 24 hour urine collection. Women with a singleton pregnancy in cephalic presentation are eligible. Excluded were women with severe pregnancy induced hypertension or preeclampsia (diastolic BP ≥ 110 mmHg, systolic BP ≥ 170 mmHg and/or proteinuria ≥ 5 gram in 24 hours), pre-existing hypertension (BP before 20 weeks of gestation ≥ 140/90 mmHg and/or using antihypertensive medication), diabetes mellitus, diabetes gravidarum requiring insulin therapy, renal disease, heart disease, HIV-seropositivity, intravenous anti-hypertensive medication, a previous caesarean section, HELLP syndrome, oliguria < 500 milliliter in 24 hours, pulmonary edema or cyanosis, fetal disorders, and abnormalities at the fetal heart rate (FHR) -monitoring are not eligible for the study.

### Procedures, recruitment, randomisation and collection of baseline data

Eligible women will be identified by the research coordinator and/or the staff of participating hospitals. These women will be referred to a research midwife or research nurse for counselling. Before entry into the study this person will explain to potential subjects the aims, methods, reasonably anticipated benefits, and potential hazards of the study. Subjects will be informed that their participation is voluntary and that they may withdraw consent to participate at any time during the study. They will be informed that choosing not to participate will not affect their care. In every centre an independent gynaecologist will be available for more detailed information both for patients and colleagues if required. After giving sufficient information written informed consent has to be obtained. The consent form must be signed before performance of any study-related activity. Patients who decide not to participate in this study will be treated according to one of the two protocols at the discretion of the attending obstetrician and analysed separately.

The study will be an open label study, as it is impossible to blind the health care workers involved for the strategy to which the woman is allocated. Cross-over between the two strategies would complicate the interpretation of study result. Although it will not be possible to prevent all cross-overs, both strategies will be performed according to strict criteria, as mentioned below.

After a patient has given informed consent for participation in the study cervical length will be measured using transvaginal sonography, and vaginal examination will be performed (Bishop score), both to assess cervical ripeness. At study entry all women will have baseline demographic, past obstetric and medical history recorded. After explanation of the study and informed consent, but prior to randomisation, we will perform a baseline measurement for quality of life (SF-36, HADS, EuroQol 6D3L) and additional questions on intervention preparedness and personal experience of the pregnancy. Subsequently, the patient will be randomised to either a policy that aims termination of pregnancy (intervention group) or a policy that aims expectant management for spontaneous delivery (expectant group). Randomisation will be performed through a web-based database which is hosted at the Academic Medical Centre (AMC) in Amsterdam. Randomisation will be 1:1 for intervention and expectant management, and it will be stratified for centre, parity and proteinuria according to the criteria above. Patients fill out additional quality of life questionnaires 6 weeks after delivery and 6 months after delivery (SF-36, HADS, EuroQoL 6D3L, SCL-90) and additional questions on personal experience of the delivery.

At local centres data-collection will be the responsibility of the local research coordinator and the regional research midwives or nurses. The data collected in this study will be coded and processed with adequate precautions to ensure patient confidentially.

### Interventions

#### Intervention group

In the **intervention group**, patients will be induced within 24 hours after randomisation. In patients with a Bishop cervix score > 6 at vaginal examination labour will be induced by amniotomy and, if needed, augmentation with oxytocin. If this score is 6 or lower cervical ripening will be stimulated with use of intracervical or intravaginal prostaglandins according to the local protocol. In case the cervix is judged to be unripe the day after 'priming', the cervical ripening will be repeated. If the cervix remains 'unripe', day 3 will be a rest day. Cervical ripening will be repeated at day 4 and 5. All patients in the intervention group will be monitored clinically until after delivery.

#### Expectant group

In **the expectant group**, patients will be monitored until the onset of spontaneous delivery. Monitoring will consist of assessment of fetal movements as reported by the mother, as well as electronic FHR-monitoring according to the local protocol. Maternal evaluation consists primarily of frequent evaluation of blood pressure measurement and screening of urine for protein using a dipstick or protein/creatinin ratio and 24 hour urine collection for protein in case of positive screening. Blood tests (platelet count, liver enzymes and renal function) will be performed according to the local protocol.

In the expectant monitoring group, intervention is recommended in case fetal condition does not justify expectant management anymore (no fetal movements reported by the mother, non-optimal FHR-monitoring). Moreover, induction of labour is recommended in case the diastolic blood pressure is ≥ 110 mmHg or the systolic blood pressure is ≥ 170 mmHg, in case 24 hours proteinuria exceeds 5 gram, in case intravenous anti-hypertensive or prophylactic anti-convulsive medication is started, in case eclampsia or the HELLP syndrome occurs. In case in the expectant group any other indication rises for induction of labour, for example prelabour rupture of membranes for > 24 hours or meconium stained liquor, patients will be induced.

### Follow up of women and infants

All details of delivery, maternal assessments and admission during pregnancy are recorded in the case record form that is accessible through the website. Maternal mortality and morbidity will be specified until date of discharge from hospital and six weeks postpartum. In case of admittance of the baby to the neonatal intensive care, high care, medium care unit or maternal ward, details of this admission are also documented. Neonatal mortality and morbidity will be specified until date of discharge from hospital. We will register the diagnosis at discharge: small for gestational age, hypoglycemia, respiratory distress syndrome, chronic lung disease, meconium aspiration, pneumothorax, apneu, asphyxia, necrotizing enterocolitis, intraventicular hemorrhage, periventricular leucomalacia, neonatal sepsis and neonatal meningitis.

A plan for long-term follow up of the mothers is in preparation. Long-term follow up of children will not be performed, because we do not expect differences between both policies during childhood.

### Outcome measures

#### Primary outcome measure

The primary outcome measure will be severe maternal morbidity, which can be complicated by maternal mortality in rare cases. Severe maternal morbidity will be defined as diastolic BP ≥ 110 mmHg, systolic BP ≥ 170 mmHg, major postpartum hemorrhage, eclampsia, HELLP syndrome, pulmonary edema, trombo-embolic disease and/or abruptio placentae [14]. Major postpartum hemorrhage is defined as blood loss > 1000 ml within 24 hours after delivery [15]. Eclampsia is defined as severe pregnancy induced hypertension or preeclampsia resulting in maternal seizures [16]. HELLP syndrome is defined as a complication of severe preeclampsia involving Hemolysis, Elevated Liver functions, and Low Platelets [17]. Trombo-embolic disease is defined as deep-vein thrombosis, pulmonary embolism or both. Patients will be examined for deep-vein thrombosis byduplex dopplerif thrombosis is suspected from clinical examination. A diagnosis of pulmonary embolism will be confirmed by pulmonary angiography, computed tomography, magnetic resonance imaging or a ventilation-perfusion lung scan [18-20].

#### Secondary outcome measures

Secondary outcomes will be neonatal mortality or neonatal morbidity, caesarean section rate, instrumental vaginal delivery rate, maternal quality of life and quality of recovery and costs. Adverse neonatal outcome will be defined as a 5-minute Apgar score below 7, an umbilical artery pH below 7.05 or admission to the neonatal intensive care.

### Statistical issues

#### Sample size

The aim of induction of labour is to reduce the rate of severe complications of hypertensive disease, such as postpartum hemorrhage, sever hypertension (diastolic BP ≥ 110 mmHg), eclampsia and HELLP syndrome. In women with a singleton pregnancy in cephalic presentation at term (>36 weeks), the prevalence of such complications in 2003 and 2004 was 12% [21]. To our opinion, the disadvantages of induction of labour outweigh the advantages when the complication rate is reduced to 6%. In order to detect such a difference, we will need two groups of 360 patients (two-sided test, alpha .05; beta .80).

#### Data analysis

The analysis will be performed by intention to treat, and stratified for centre, parity and for underlying disease (preeclampsia or pregnancy induced hypertension). Quality of life as well as pain scores will be analysed using repeated measures analysis of variance [22]. Relative risks and 95% confidence intervals will be calculated for the relevant outcome measures.

Moreover, we will evaluate whether the relative benefits of induction of labour will be stronger in women with a ripe cervix at baseline and in women with a short cervical length at transvaginal sonography. In case of equivalence between outcomes, the analysis will be repeated on a par protocol basis.

### Economic analysis

The process of care is distinguished into three cost stages (antenatal stage, delivery/childbirth, postnatal stage) and three cost categories (direct medical costs [all costs in the health care sector], direct non-medical costs [costs outside the health care sector that are affected by health status or health care] and indirect costs of the pregnant women and her partner [costs of sick level]). For each stage and each cost category, costs are measured as the volumes of resources used multiplied with appropriate valuations (cost-per-unit estimates, fees, national reference prices). Cost volumes in the antenatal stage consist of direct medical costs (e.g. home/hospital care, outpatients' visits, fetal monitoring [FHR-monitoring, ultrasound, Doppler] and maternal monitoring [various labtests; hospital care]). Direct non-medical and indirect costs in that stage may occur if role patterns or household routines shift. As we anticipate an improvement of between maternal outcomes after induction of labour the economic analysis is expected to be a cost-effectiveness analysis.

**Serious adverse events **will be reported to an independent data safety monitoring committee. A formal interim analysis is not planned.

**Ethical considerations **The study protocol has been approved by the Medical Ethical Committee of the University Medical Centre of Leiden (p04.210). The protocol is registered in the clinical trial register number ISRCTN08132825.

## Discussion

Pregnancy induced hypertension and preeclampsia are important hypertensive disorders during pregnancy which are associated with increased maternal and neonatal morbidity and mortality. There is no consensus on how to manage mild hypertensive diseases at term. Induction of labour might prevent maternal complications, but is also thought to increase the caesarean and vaginal instrumental delivery rate. This trial is designed to provide evidence on the effectiveness of induction of labour in women with mild pregnancy induced hypertension or preeclampsia (nearly) at term to prevent severe maternal and neonatal complications.

## Abbreviations

BP – blood pressure

FHR – fetal heart rate

HELLP – Hemolysis Elevated Liver enzymes Low Platelets

AMC – Academic Medical Centre

## Competing interests

The author(s) declare that they have no competing interests.

## Authors' contributions

MvP and BWM were involved in conception and design of the study. CK, MvP and BWM drafted the manuscript. All authors mentioned in the manuscript are member of the Hypitat study group. They participated in the design of the study during several meetings and are local investigators at the participating centres. All authors edited the manuscript and read and approved the final manuscript.

## Pre-publication history

The pre-publication history for this paper can be accessed here:


